# Supine Bridge Exercise in Degenerative and Functional Hip Disorders: A Biomechanical and Therapeutic Approach (Part III)

**DOI:** 10.7759/cureus.85678

**Published:** 2025-06-10

**Authors:** Saverio Colonna, Riccardo Tarozzi, Antonio D'Alessandro, Fabio Casacci

**Affiliations:** 1 Rehabilitation Medicine, Spine Center, Bologna, ITA; 2 Research and Development, Osteopathic Spine Center Education (OSCE), Bologna, ITA

**Keywords:** cam-type fai, femoral anterior glide syndrome, femoroacetabular impingement, gluteus maximus activation, hip disfunction, hip joint stability, hip pathologies, microinstability of the hip, synergistic dominance, upine bridge exercise

## Abstract

The supine bridge exercise (SBE) is widely recognized in rehabilitation for improving core stability and hip extensor strength. While its benefits in low back pain have been documented, its role in hip joint dysfunctions remains underexplored. This narrative review investigates the application of the SBE in degenerative and functional hip disorders, including femoroacetabular impingement (FAI), microinstability, and femoral anterior glide syndrome (FAGS). Particular attention is given to the biomechanical rationale behind gluteus maximus activation (especially the lower portion, or LGM) and the inhibition of synergistic dominance by the hamstrings Based on current evidence, specific SBE variations, including hip and ankle positioning, spinal alignment, and neuromuscular control strategies, may promote posterior femoral head translation and joint stability. Furthermore, the review highlights how in specific athletic populations, such as soccer players and dancers (where cam-type FAI alterations and restricted hip internal rotation are particularly prevalent), the inclusion of SBE sessions into preventive training programs could contribute to preserving hip joint health and mitigating degenerative processes. We argue that SBE when appropriately tailored, can become a fundamental therapeutic tool in both conservative management and functional retraining of hip dysfunctions.

## Introduction and background

The use of the supine bridge exercise (SBE) has garnered increasing attention in the clinical rehabilitation of various musculoskeletal disorders [1-3]. Following previous articles on the execution modalities of the SBE [4] and its application in low back pain [5], this third installment of the series narratively explores its therapeutic relevance in hip pathologies. This narrative review focuses on the anatomical, biomechanical, and neurophysiological aspects that support the use of the SBE in conservative treatment protocols for non-traumatic hip dysfunctions and pathologies.

## Review

Hip joint pathology

Degenerative hip disease, known as coxarthrosis, is characterized by progressive wear of the articular cartilage, leading to pain and reduced mobility. The causes of this condition can be divided into two main categories: primary and secondary [6]. Primary coxarthrosis develops without an identifiable cause and is mainly associated with aging. As age advances, cartilage may undergo degeneration, leading to primary coxarthrosis. Secondary coxarthrosis results from specific factors that negatively affect the hip joint, including hip dysplasia, fractures, avascular necrosis of the femoral head, femoroacetabular impingement, and obesity [7]. Understanding these causes is crucial for early diagnosis and effective management of coxarthrosis to prevent progression, alleviate pain, and improve patients’ quality of life.

Therapeutic exercise has been cited as an important approach in the management of hip osteoarthritis [8-10]; however, there is a notable lack of literature investigating the type and effectiveness of therapeutic exercise for this condition. Existing programs are often quite generalized, with short-term effects ranging from small to moderate, and poorer long-term outcomes [10-13].

The development of more targeted programs, based on a deeper understanding of the function and dysfunctions involving the hip joint, could improve clinical outcomes.

Femoroacetabular impingement (FAI)

FAI is a pathological condition of the hip in which abnormal contact occurs between the femoral head and the acetabular rim during joint movements, particularly during flexion and internal rotation [14]. This abnormal contact can cause pain and, over time, damage intra-articular structures such as the acetabular labrum and cartilage.

There are two main forms of FAI: the "cam" type, caused by a bony prominence at the femoral head-neck junction that impinges against the acetabular rim; and the "pincer" type, characterized by excessive coverage of the femoral head by the acetabulum [14]. A mixed type also exists, representing a combination of both mechanisms [15].

FAI is often observed in young and physically active individuals and may be associated with joint motion limitations, groin pain, and if left untreated, progression to joint degeneration.

Cam-type FAI

**Figure 1:**
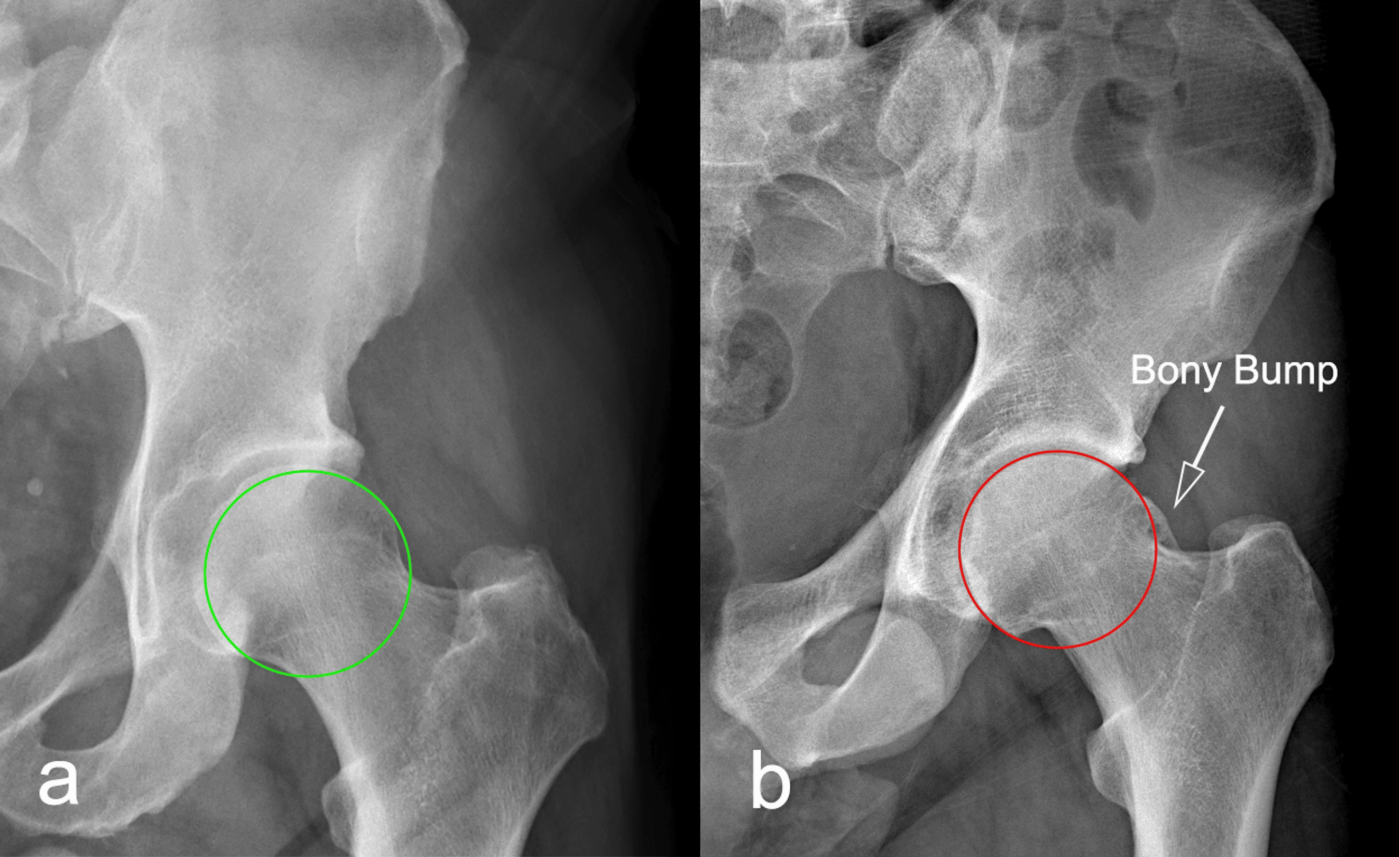
Coronal plane radiographs of hip a) Normal hip joint; b) hip joint showing a clear superior head-neck deformity, defined as a bony bump or pistol grip deformity. Image credit: Author, Saverio Colonna

**Figure 2:**
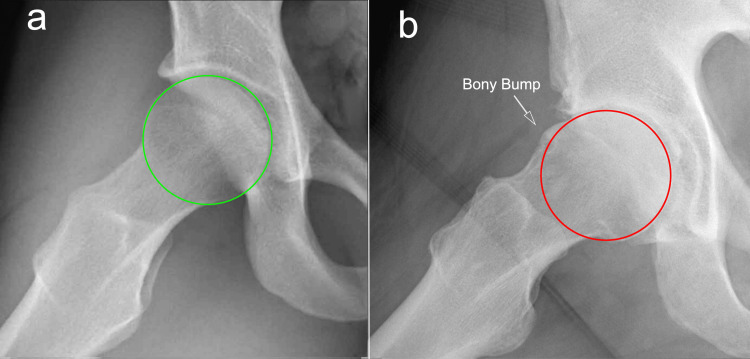
Dunn view radiographs of hip a) Normal hip joint; b) hip joint showing a clear anterior head-neck deformity, defined as a bony bump. Image credit: Author, Saverio Colonna

Cam deformity refers to a non-spherical prominence of the anterosuperior portion of the femoral head (Figures 1, 2), often described as a “bony bump” [15] or “pistol grip” deformity [6,16].

During hip motion, this prominence can impinge against the acetabular rim, resulting in the so-called cam impingement [14]. This condition is frequently associated with hip pain, limited internal rotation, acetabular labral tears, and cartilage damage, and, over time, may progress to hip osteoarthritis [17,18].

The management of cam deformity and associated FAI remains subject to ongoing discussion. While hip arthroscopy has demonstrated effectiveness in improving outcomes in symptomatic patients [6,17], its role in asymptomatic or borderline cases is less clear. As physical therapy cannot modify the underlying bony morphology, it may still play a valuable role by influencing pelvic mechanics, particularly in mild to moderate presentations [19,20].

According to some authors [21], instability in cam-type FAI appears to be primarily anterosuperior. In the altered position of the femoral head, migrated more anteriorly within the acetabulum, the contact area between the femoral head and acetabular cartilage is significantly reduced compared to the physiological position. As a result, compressive and shear forces are markedly increased. It is well established that cartilage poorly tolerates shear forces, which are considered particularly damaging [22].

A deeper understanding of microinstability is clinically relevant, as anterosuperior translation of the femoral head is thought to be harmful to hip joint health due to the inherently reduced congruency of the articulation-a condition fundamentally related to pelvic retroversion stemming from human evolution [23].

An intact hip joint possesses a high degree of intrinsic multiplanar mobility. Stabilizing structures include the acetabular labrum, ligamentum teres, iliofemoral, ischiofemoral, and pubofemoral ligaments, the joint capsule [24,25], the iliopsoas tendon [26], the rectus femoris tendon [27], and the iliocapsularis muscle [28].

The anterior capsule and acetabular labrum are especially critical in resisting the anterior translation of the femoral head [29,30]. Ligamentous laxity and weakness of the peripelvic musculature can contribute to the manifestation of hip microinstabilities [31]. The hip joint position that places the greatest tension on the anterior capsule is maximal extension combined with external rotation [26,32-34].

This position is used as a pain and/or apprehension provocation test in cases of hip instability and is therefore referred to as the "apprehension hip test" [24,35,36], analogous to the shoulder apprehension test [37].

Physical activity, hip dysfunction, and cam-type FAI

The hip joint plays a critical role in athletic performance and is therefore susceptible to injury [38]. Groin and hip injuries are common among athletes, particularly in sports involving kicking, sudden changes of direction, twisting, pivoting, and rapid acceleration and deceleration [39]. According to Sahrmann, even long-distance runners may be at increased risk of anterior femoral head translation due to the exaggerated hip extension inherent in running [40]. This mild instability, combined with repetitive hip extension, may contribute to labral tears [41].

To further explore the relationship between dysfunction type and atraumatic hip pathology, we present two examples of physical activity (soccer and dance) which, although very different in nature, share a high incidence of hip pain and restricted internal rotation. This may be due to the anterior translation of the femoral head associated with specific technical movement patterns.

It has been suggested that shortening of the myofascial systems of the hip abductors and external rotators-manifested as reduced internal rotation-may contribute to functional alterations which, over time, could play a role in the progression of degenerative changes in the hip joint [42].

This is further supported by the following discussion of soccer and dance, both of which are associated with restricted internal rotation and a high incidence of FAI. While the precise causes of restricted internal rotation remain debated, both neuromuscular factors (such as hyperactivation of the abductor-external rotator group) and structural adaptations resulting from mechanical stress during growth may play a role.

Soccer and Cam-Type FAI

Hip and groin pain account for 11% to 16% of injuries leading to time loss in soccer [43]. Since soccer players frequently report symptoms related to the hip or groin [44], injuries classified strictly as “time-loss” likely represent only the more severe cases [45]. Thus, relying solely on time-loss definitions probably underestimates the true incidence of hip and groin pain in soccer players.

A recent study involving 120 professional soccer players and 80 non-athlete controls found radiographic evidence of cam-type FAI in 61.6% of the soccer players and only 11.6% of the control group [46]. Internal hip rotation is one of the first movements found to be limited in youth athletes with cam-type FAI, as shown in longitudinal follow-up studies (2-5 years) [47,48].

Some authors report a strong association between limited internal hip rotation and hip pain, but not between limited rotation and the presence of cam deformity [49]. This suggests that cam deformity may not be the primary cause of restricted motion. Notably, decreased hip range of motion (particularly internal rotation) has been associated with increased hip and groin symptoms and prior injuries in professional soccer players, even in the absence of a cam deformity.

Male soccer players typically show greater limitations in internal rotation than female soccer players [50], a feature that may reduce the risk of anterior cruciate ligament injury but increase susceptibility to cam-type FAI.

This view is not supported by all studies; some authors [51,52] find a stronger association between hip pain and limited hip flexion rather than limited internal rotation.

Some researchers [47,48] attribute the “pistol grip” deformity of the femoral head-neck junction to excessive loading in individuals with incompletely closed proximal femoral growth plates.

In a large study of human skeletal remains, Goodman et al. found a prevalence of 8% for pistol grip deformity, with a higher incidence of severe osteoarthritis in the affected group compared to the unaffected group (38% vs. 26%) [52]. The authors proposed that this deformity could be the result of subclinical slipped capital femoral epiphysis and a possible risk factor for hip osteoarthritis. However, Resnick, who observed this deformity in 48 of 100 patients undergoing hip surgery for osteoarthritis, concluded it was primarily the result of bony remodeling and osteophyte formation, rather than prior epiphyseal slippage [53]. While some authors [47,48] link the deformity to excessive loading in skeletally immature individuals, others [18,54] suggest it may be secondary to osteoarthritis in terms of severity and frequency. Notably, decreased hip range of motion (particularly internal rotation) has been associated with increased hip and groin symptoms and prior injuries in professional soccer players, even in the absence of a cam deformity.

Based on the literature, it cannot be ruled out that the bone alteration characteristic of the pistol grip deformity seen in cam-type FAI may itself be secondary to chronic friction between the femoral head-neck and capsuloligamentous structures or the labrum, driven by abnormal anterior femoral head translation.

Chronic mechanical stress significantly affects the structure and tissue properties of bone, particularly during skeletal development [55]. Immature skeletons are especially responsive to mechanical loading, due to their greater tissue elasticity and more active remodeling processes [56].

One study found that pistol grip deformity is more frequent in male individuals [18]. This is consistent with the observation that male individuals have a greater tendency than female individuals to exhibit hyperactivation of the hip external rotators, which, along with other osseous features of pelvic morphology [18], contributes to internal rotation limitation in both general and athletic populations [57,58].

Dance and Cam-Type FAI

Dance, especially classical ballet, is a predominantly female activity; yet, dancers exhibit a higher incidence of hip pain [59,60] compared to the general population. Additionally, professional dancers have a higher rate of hip and/or groin injuries than amateur dancers [60].

In one study, of 82 professional ballet dancers (44 women and 38 men), isolated cam-type FAI was diagnosed in 106 hips (65%), isolated pincer-type FAI in six hips (3.6%), and mixed-type FAI in seven hips (4.2%) out of 164 hips [61]. Only 49 hips (29.5%) (less than one-third) showed no osseous abnormalities. Male dancers had a higher relative risk for cam-type FAI than female dancers [61].

The aesthetic requirements of dance, particularly classical ballet, often demand a "perfect turnout," or 180° of external rotation of the lower limbs. Ideally, most of this rotation should originate from the hip joint. Literature indicates that of the ideal 90° of unilateral external rotation, approximately 60°-70° comes from the hip, with the remaining 10°-35° from distal segments [62].

MRI assessments comparing dancers to non-dancers have revealed greater mean femoral head subluxation (2.05 mm; range 0.63-3.56 mm) in dancers, which authors identified as a contributing factor to increased joint degeneration [63].

A study comparing senior and elite female ballet dancers with age-matched controls found reduced hip internal rotation and adduction among dancers [64,65]. The authors attributed this to overuse of the hip abductor-external rotator muscles, with insufficient stretching and underuse of adductor-internal rotator muscles. Therefore, they recommend regular stretching of tight myofascial structures for injury prevention [59,66].

In a case report, conservative treatment aimed at reducing anterior femoral head translation led to the resolution of hip pain in a dancer with suspected labral injury [67].

Hip microinstability and femoral anterior glide type

Hip microinstability has recently gained recognition as a distinct clinical entity [68-71], although objective, evidence-based diagnostic criteria are still lacking. It is defined as painful supraphysiologic hip motion associated with structural and functional abnormalities that compromise joint stability [25], primarily through altered biomechanics. As a result of abnormal kinematics between the femoral head and the acetabulum, surrounding structures such as the labrum, capsule, and cartilage may be subjected to excessive stress. Hip microinstability, through these altered forces, can lead to labral tears even in the absence of traumatic events [72,73], potentially contributing to early degenerative changes [73]. The most frequently involved labral region in such injuries is the anterosuperior quadrant [74,75].

**Figure 3:**
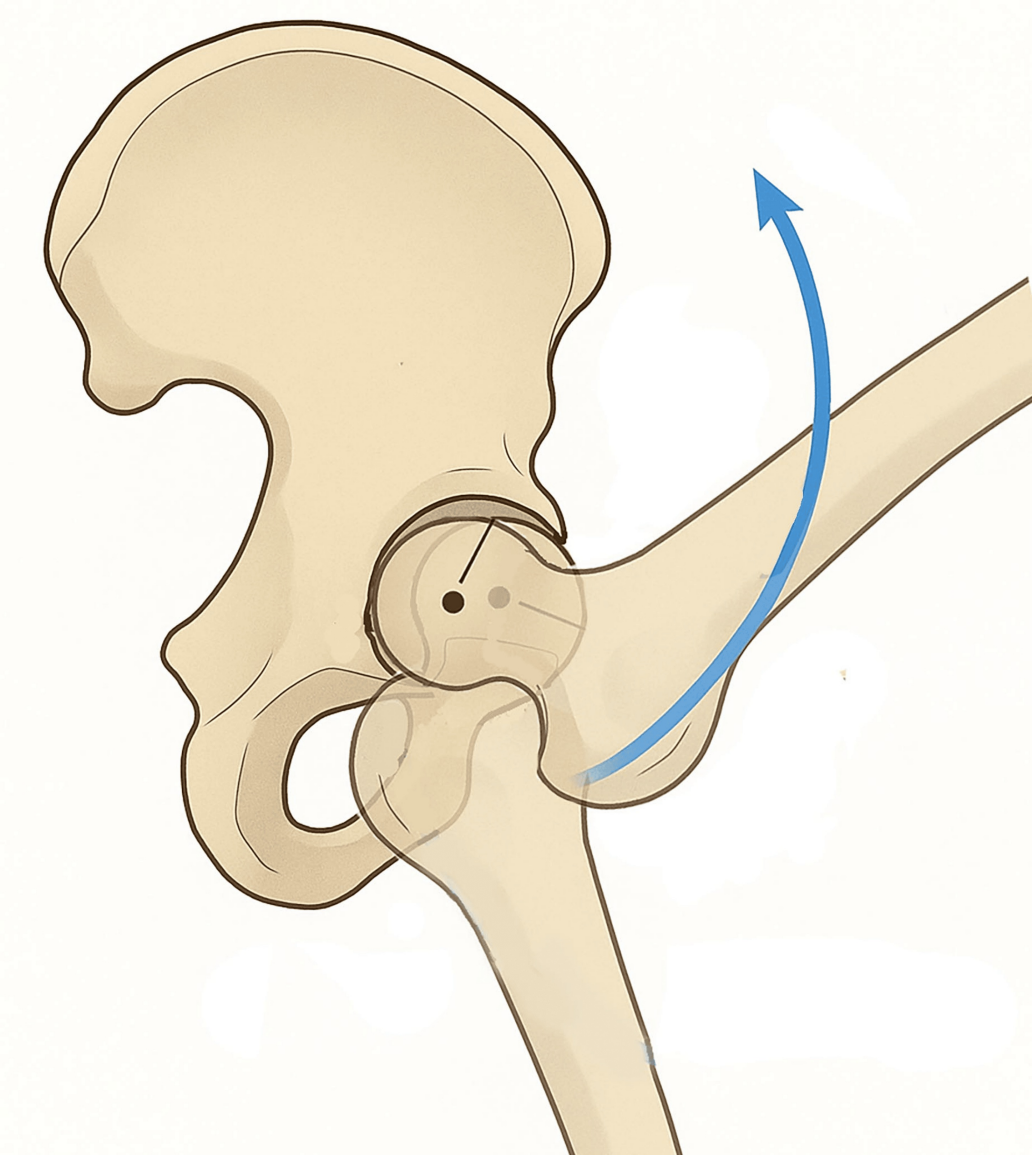
Schematic representation of hip flexion In evidence physiological posterior glide of the center of rotation during hip flexion. Image credit: Author, Saverio Colonna

According to Sahrmann, one of the primary causes of hip joint dysfunction is excessive anterior glide combined with lateral rotation of the femoral head, described as the femoral anterior glide type (FAGT) [40]. This pattern is characterized by excessive anterior translation or insufficient posterior glide of the femoral head during hip flexion [40,76]. In normal hip flexion, the femoral head performs a slight posterior glide (Figure 3) to maintain the stability of the instantaneous center of rotation (PICR) [77-79].

In patients with FAGT, this posterior translation is impaired, resulting in altered joint mechanics and contributing to FAI with anterior hip pain.

Sahrmann and Lewis et al. suggest that this altered movement may arise from weakness or insufficient activation of the iliopsoas during flexion and/or the gluteal muscles during extension [40,80]. Over time, this pattern may lead to overstretching of passive stabilizing structures and an anterosuperior shift of the PICR. Although direct measurement of anterior femoral head glide during active hip motion in vivo has not yet been accomplished, existing evidence supports the plausibility of this motor pattern as a contributor to hip pain [81]. MRI studies of dancers in neutral and front split positions revealed an average subluxation of the femoral head of 2.05 mm (range 0.63-3.56 mm) in extension [63].

Cadaveric studies have also demonstrated anterior glide of the femoral head within the acetabulum in response to posterior-to-anterior mobilization forces, with displacements ranging from 0.25 to 2.90 mm [82]. Conversely, posterior glide in healthy subjects during manual mobilization has been measured via ultrasound at a mean of 2.0 mm [83], suggesting that although the hip is a congruent joint, translational movements of the femoral head do occur under load.

Patients with FAGT often display characteristic motor patterns. During active straight leg raises in the supine position, the anterior or anteromedial motion of the greater trochanter is visible or palpable and frequently accompanied by groin pain [40]. Similarly, during prone hip extension, the greater trochanter again moves anteriorly or anteromedially rather than maintaining a stable position.

Sahrmann notes that excessive internal (or medial) rotation may be driven by dominant activation of the tensor fasciae latae, a muscle that contributes to both hip flexion and internal rotation, rather than appropriate use of the iliopsoas [40]. Induction of internal rotation in the context of a shifted PICR may result in abnormal compression of the labrum, potentially leading to labral damage.

Labral injury is considered a primary event in the cascade leading to hip osteoarthritis [84]. As the labrum contains nociceptive endings [85], its involvement may contribute to early inhibition of internal rotation [86,87], which is frequently the first movement to become limited in symptomatic hips. Notably, most clinical tests for labral pathology, such as the FADIR (Flexion, Adduction, Internal Rotation) test, provoke symptoms through internal rotation and adduction in a flexed hip position [33,88].

FAGT and muscular dysfunction

In both research and clinical practice, little attention has been paid to the functional differentiation of the gluteus maximus (GM) muscle and its impact on hip joint biomechanics and the implications for preventive and rehabilitative exercise prescriptions targeting hip osteoarthritis. The GM is composed of two functionally distinct regions: the upper portion (UGM), originating from the posterior iliac crest and inserting (along with the tensor fasciae latae, or TFL) onto the iliotibial band (ITB), and the lower portion (LGM), originating from the lower sacrum, coccyx, sacrotuberous ligament, posterior sacroiliac ligaments, and the fascia covering the multifidus muscle [89,90].

Although there is no distinct fascial separation between these regions in adults, developmental studies have shown that the GM derives from two myogenic primordia that remain separated by loose connective tissue in the fetus before fusing during the prenatal period [91]. The force vector of the UGM passes above the center of rotation of the hip, providing a primary function in hip abduction and external rotation [92]. While both portions can contribute to hip extension, the LGM, acting below the center of rotation, serves as the primary extensor [90,93,94] and plays a crucial role in absorbing ground reaction forces during heel strike in gait.

**Figure 4:**
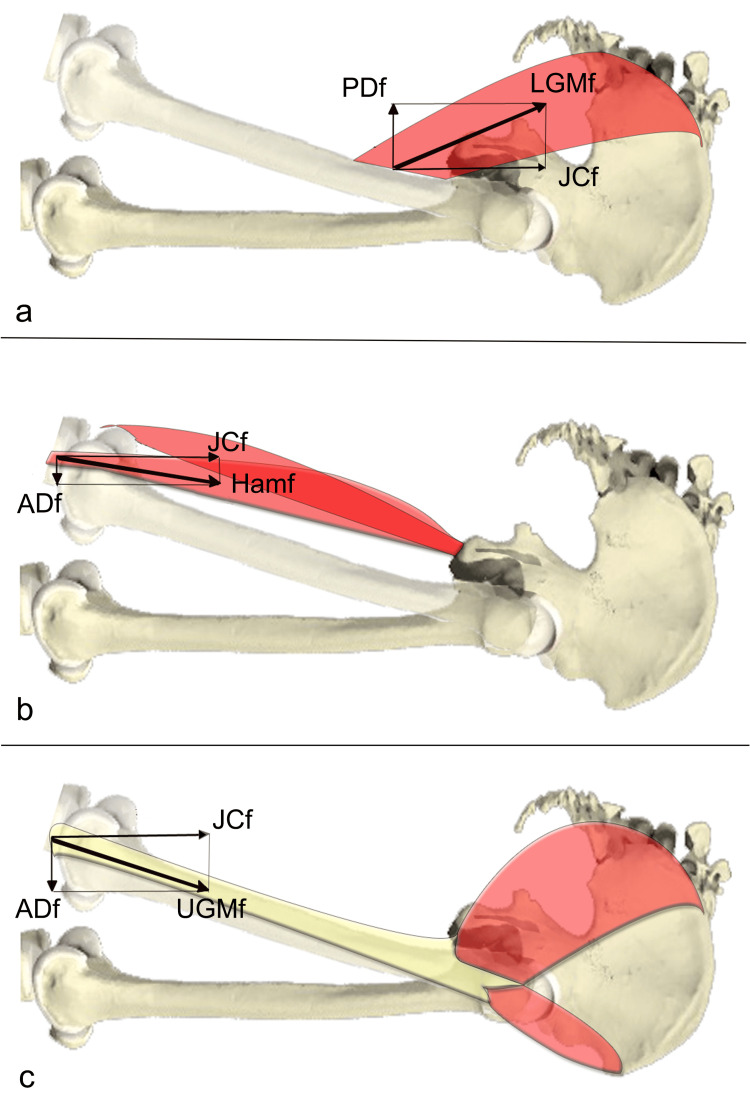
Biomechanical diagram of the force exerted by gluteus maximus and hamstrings a) Lower gluteus maximus force (LGMf), divided into the force vectors expressed in the sagittal plane: JCf and PDf posteriorly directed force; b) hamstrings (Hamf), divided into the two force vectors expressed in the sagittal plane: JCf and ADf; c) upper gluteus maximus (UGMf), divided into the force vectors expressed in the sagittal plane: JCf and ADf. JCf: joint compression force; PDf: posteriorly directed force; ADf: anteriorly directed force Reprinted with permission from [42].

Anterior femoral head glide (FAGT) is commonly attributed to excessive recruitment of the hamstrings relative to the GM during hip extension. As shown in a biomechanical analysis [42], the hamstring force vector includes an anteriorly directed component that tends to translate the femoral head forward, whereas LGM activation generates a posteriorly directed force. Specifically, the LGM contributes a joint compression force (JCf) that stabilizes the femoral head in the acetabulum, and a posteriorly directed force (PDf) orthogonal to the former, acting to limit anterior translation (Figure 4a). In contrast, the hamstring force vector includes a cranially directed JCf and an anteriorly directed force (ADf) that promotes anterior translation (Figure 4b). The same applies to the UGM, where a joint compression force vector (JCf) and an anteriorly directed force vector (ADf) are present (Figure 4c).

It must be noted, however, that the LGM comprises only about 20% of the total GM volume [40]. Although the UGM is considered the dominant portion [95] and may also produce an anteriorly directed vector similar to the hamstrings (especially through its tibial ITB insertion), its femoral insertion onto the lateral intermuscular septum may contribute to posteriorly directed forces [96,97]. Therefore, imbalances not only between hamstrings and GM but also between the UGM and LGM may significantly influence the direction of hip joint reaction forces.

Our current focus is on the imbalance between the two portions of the GM, as well as between the hamstrings and GM while being aware, as emphasized by a recent study [98], of the important role played by the deep periarticular muscles of the hip joint.

Maintaining the physiological instantaneous center of rotation (PICR) during hip extension requires early and predominant activation of the LGM over the hamstrings [99,100]. Unfortunately, it remains unclear whether the two GM portions can be selectively activated. Anatomically, the inferior gluteal nerve (L5-S2) branches into medial, lateral, and inferior divisions upon reaching the GM, but it is not known whether these divisions innervate the UGM and LGM separately [101]. If it were established that the two portions of the gluteus maximus have distinct innervations, there could be grounds for distinct action, as well documented in the literature [90,102].

Rehabilitation exercises focused on the posteriorization of the femoral head, in the case of anterior dysfunction, should be specifically aimed at activating the LGM component.

**Figure 5:**
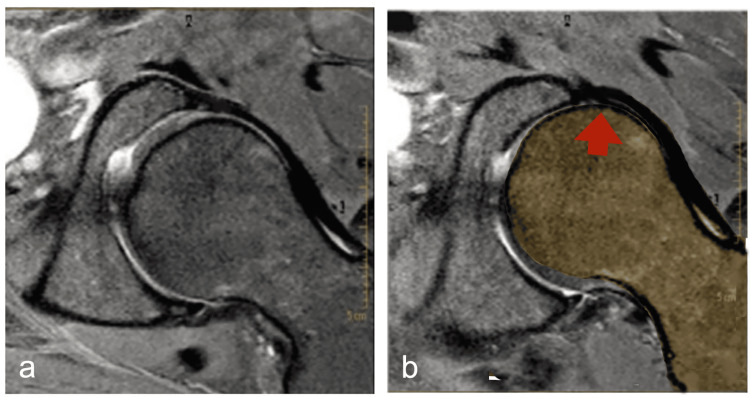
MRI image of the hip showing schematization of femoral head within the acetabulum a) Physiological; b) dysfunctional anterior glide of the femoral head with increased tension of the anterior stabilizing structures. Image credit: Author, Saverio Colonna

From our perspective, it is the hyperactivation or shortening of the hip extensors-abductors-external rotators (such as the UGM) that drives the femoral head anteriorly, leading to microinstabilities and loss of physiological joint rotation centers [79] (Figure 5). This anterior shear places excessive tensile stress on anterior structures such as the labrum, iliofemoral, and pubofemoral ligaments, and the iliopsoas and rectus femoris tendons.

Such mechanisms may underlie some cases of so-called idiopathic degenerative changes, while others with structural abnormalities (e.g., Legg-Calvé-Perthes disease, hip dysplasia, slipped capital femoral epiphysis, or femoroacetabular fractures) follow different biomechanical patterns. Whether UGM hyperactivation is a primary cause or a secondary effect of degeneration remains uncertain. Some authors argue that even when secondary, increased activation of the GM elevates compressive loading across the articular surfaces and may accelerate degeneration [103]. Notably, acetabular peak pressure has been shown to correlate more closely with peak GM electromyographic activity than with peak ground reaction forces [104], prompting some authors to caution against strengthening the hip abductors.

Other studies emphasize the importance of evaluating the UGM and LGM independently [105]. Magnetic resonance imaging studies have shown significant volume reductions in both UGM and LGM on the affected side in patients with advanced hip osteoarthritis. However, when comparing patients to healthy controls, no significant difference in LGM volume was found, whereas UGM volume was significantly larger in patients. No differences were observed for the TFL. These findings suggest that asymmetries in GM volume may be partly due to atrophy on the affected side, but also to compensatory hypertrophy on the contralateral side in response to antalgic weight-shifting strategies.

Specifically, the LGM on the unaffected side in advanced cases was 15.2% larger than in matched controls, although this difference did not reach statistical significance. Conversely, the UGM volume on the unaffected side was significantly larger (mean difference: 30.5%) than in controls, suggesting that the 21% asymmetry observed in advanced hip pathology may be largely due to contralateral hypertrophy. Some degree of atrophy on the affected side cannot be excluded, although fatty infiltration of the UGM is not commonly reported on MRI.

The sacral fibers of the GM, especially the deep fibers, run perpendicularly to the sacroiliac joint and contribute to its stability, playing a key role in force transmission from the lower limb to the pelvis during gait [106,107]. However, the GM is often weak and elongated due to prolonged sitting [40], a condition that can lead to sacroiliac joint dysfunction and low back pain [108]. Increased hamstring tension is frequently observed as a compensatory mechanism for weak GM function [108,109]. Excessive anterior pelvic tilt, dominant erector spinae activity, and lumbar hyperlordosis may also result from GM weakness or delayed activation during hip extension [40].

Hypotheses on the neurophysiological mechanisms underlying myofascial dysfunctions in FAGT

Understanding the causes of gluteus maximus (GM) dysfunction is essential for identifying effective corrective strategies aimed at preventing and/or reducing the risk of degenerative joint lesions. One key factor appears to be lifestyle, which can negatively influence GM activation. In particular, prolonged sitting may impair GM stimulation, leading over time to muscle weakening and progressive atrophy [89].

Synergistic Dominance

GM weakness is believed to result from increased reliance on secondary hip extensors-such as the hamstrings and hip adductors-to generate hip extension torque [110]. This neurophysiological phenomenon is referred to as synergistic dominance [40].

Synergistic dominance describes a condition in which a primary agonist muscle group becomes dependent on a secondary agonist group due to weakness and/or restriction of the former (e.g., hamstrings compensating for GM) [40,111-114]. These neuro-myofascial alterations may cause hyperactivity of the secondary synergistic muscles to compensate for weak or inhibited primary movers [112,113,115,116].

Reciprocal Inhibition

To better understand the rationale for targeting specific muscles in managing hip osteoarthritis, another neurological mechanism must be introduced: reciprocal inhibition. This reflex governs the physiological relationship between agonist and antagonist muscle groups [117]. It is based on the length-tension relationship, which must be preserved to prevent inhibition of the antagonist group; for example, myofascial tightness in the hip flexors can inhibit their antagonists (the hip extensors) including the GM [111,118].

When reciprocal inhibition occurs, a neuromuscular dysfunction may emerge, triggering a movement syndrome often associated with myofascial imbalances and altered neuromuscular activity around a joint. Both of the aforementioned neurophysiological mechanisms have been observed between the UGM and the gluteus medius (synergistic dominance), and between the gluteus medius and the hip adductor group (reciprocal inhibition) [102]. McAndrew et al. hypothesized segmental functional differences within the GM muscle, specifically between the UGM and LGM [102]. Due to fiber orientation, the UGM behaves more like an abductor in synergy with the gluteus medius, while the LGM acts as the primary hip extensor.

Additionally, it can be theorized that limited hip flexor length reduces the neural drive to the hip extensors via reciprocal inhibition. More specifically, it has been proposed that GM inhibition may be secondary to hyperactivity or tightness of the hip flexor group, potentially leading to pathological conditions [114,119].

This inhibition could, via the mechanism of synergistic dominance [40], increase dependence on the hamstrings (secondary hip extensors) to generate adequate hip extension torque [110]. Reliance on secondary extensors may lead to increased tissue stress in the hamstrings and adductor muscles, raising the risk of soft tissue injuries (e.g., muscle strains) [120]. However, current literature lacks robust evidence to support the clinical theory that reduced hip flexor length is an underlying factor contributing to altered neuromuscular control of the lower limbs.

The aim of the study by Mills et al. was to compare lower-limb strength, muscle activation, and biomechanics between individuals with and without limited hip flexor length [113]. The primary hypothesis was that individuals with reduced hip flexor flexibility would exhibit, compared to those with normal flexibility, lower hip extension strength and greater internal knee extension moment during the descent phase of a bilateral squat. The secondary hypothesis was that those with shorter hip flexors would also demonstrate reduced GM activation and increased activation of the biceps femoris compared to those with normal length.

The study's findings, based on an examination of 40 female soccer athletes (20 with normal and 20 with reduced hip flexor flexibility), revealed that participants with reduced flexibility exhibited similar hip and knee extension moments as those with normal muscle length. However, they achieved these moments with reduced GM activation and greater relative co-activation of the hamstrings. This diminished GM activation may be attributed to reciprocal inhibition caused by hip flexor tightness.

These results suggest that increased stiffness of the hip flexor muscles may be an important factor to consider in injury prevention programs for knee tendons or ligaments. Consequently, the authors recommend that clinicians consider implementing treatments aimed at improving hip extension range of motion and strengthening the LGM [121], the agonist to the hamstrings.

Achieving and maintaining the physiological length of the hip flexor muscles may thus indirectly reduce hamstring hyperactivation through the neuromuscular inhibitory control exerted over the LGM.

Therapeutic exercise and FAGT

Several articles in the literature provide insights into how FAGT can be addressed with therapeutic exercise. In a case report, for example, the treatment of a female subject with FAGT was described [122]. The program, which lasted for five weeks, included four exercises: quadruped rock back, side-lying hip abduction and external rotation, prone hip extension, and sitting knee extension. The patient, exhibiting excessive internal rotation, justified the inclusion of exercises to strengthen the abductor/external rotator muscles. However, there is an evident contradiction in the program, as noted by the authors, where the sitting knee extension exercise was used to stretch the hamstrings, while the prone hip extension exercise tended to activate the hamstrings [123].

Khoo-Summers and Bloom [67] described the case of a 29-year-old professional dancer with suspected labral tear and femoral anterior glide syndrome, diagnosed according to the criteria proposed by Sahrmann [40]. The patient complained of anterior hip pain and exhibited positive signs in hip extension tests in the prone position, active leg raising in the supine position, and the quadruped rocking test. However, orthopedic tests were also positive, suggesting the presence of a labral tear.

The treatment focused on reducing anterior joint stress and improving motor control through the correction of alignment and movement alterations observed during functional activities and dance execution. After six sessions over two months, the patient reported complete resolution of pain during daily activities and most dance movements.

At the five-month follow-up, the hip pain was fully resolved, and the dancer had returned to her role as the lead dancer in a ballet company. This case highlights the importance of assessing and treating movement disorders, even in the presence of structural pathologies such as labral tears.

In another study, a case series of three elite athletes diagnosed with femoral anterior glide syndrome were treated conservatively using manipulation, therapeutic exercise, and microcurrents, with the primary goal of promoting posterior femoral head glide [124]. The rehabilitation program included exercises aimed at developing independent hip joint control and improving dissociation between hip and lumbar spine movements. The results in all three cases were excellent.

SBE and FAGT

As previously discussed, selective activation of the LGM through specific SBE variations can help posteriorize the femoral head during extension, reducing anterior shear forces associated with FAGT. This makes the SBE a targeted intervention to restore physiological hip mechanics and improve joint stability. The rationale for this approach, including the biomechanical distinctions between LGM and other hip extensors, has been presented in the previous section.

**Figure 6:**
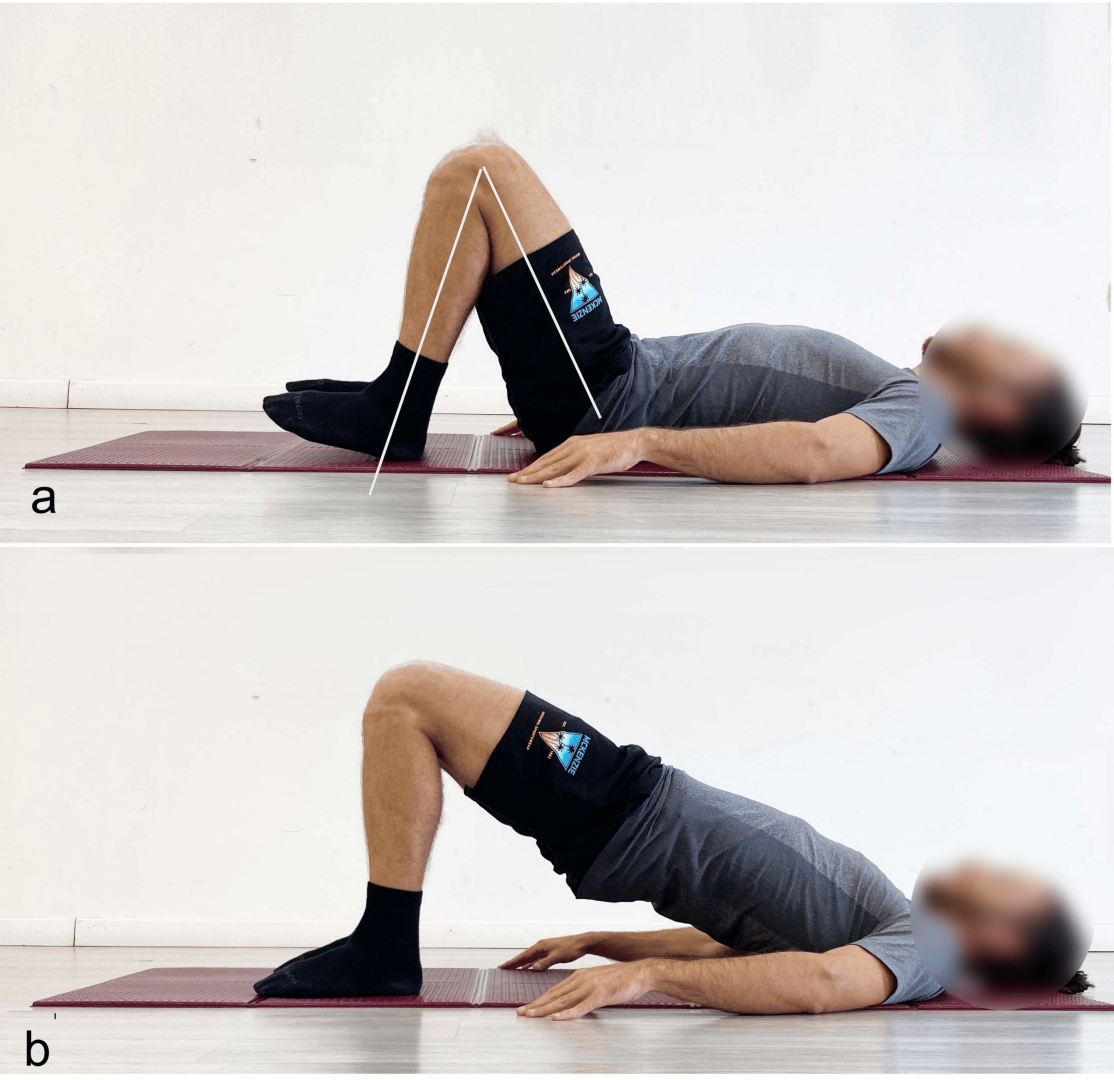
Example of supine bridge exercise performed with high degrees of knee and hip flexion in the starting position a) Starting position; b) end position. Reproduced from Colonna et al. [4], under a Creative Commons Attribution License (CC BY).

**Figure 7:**
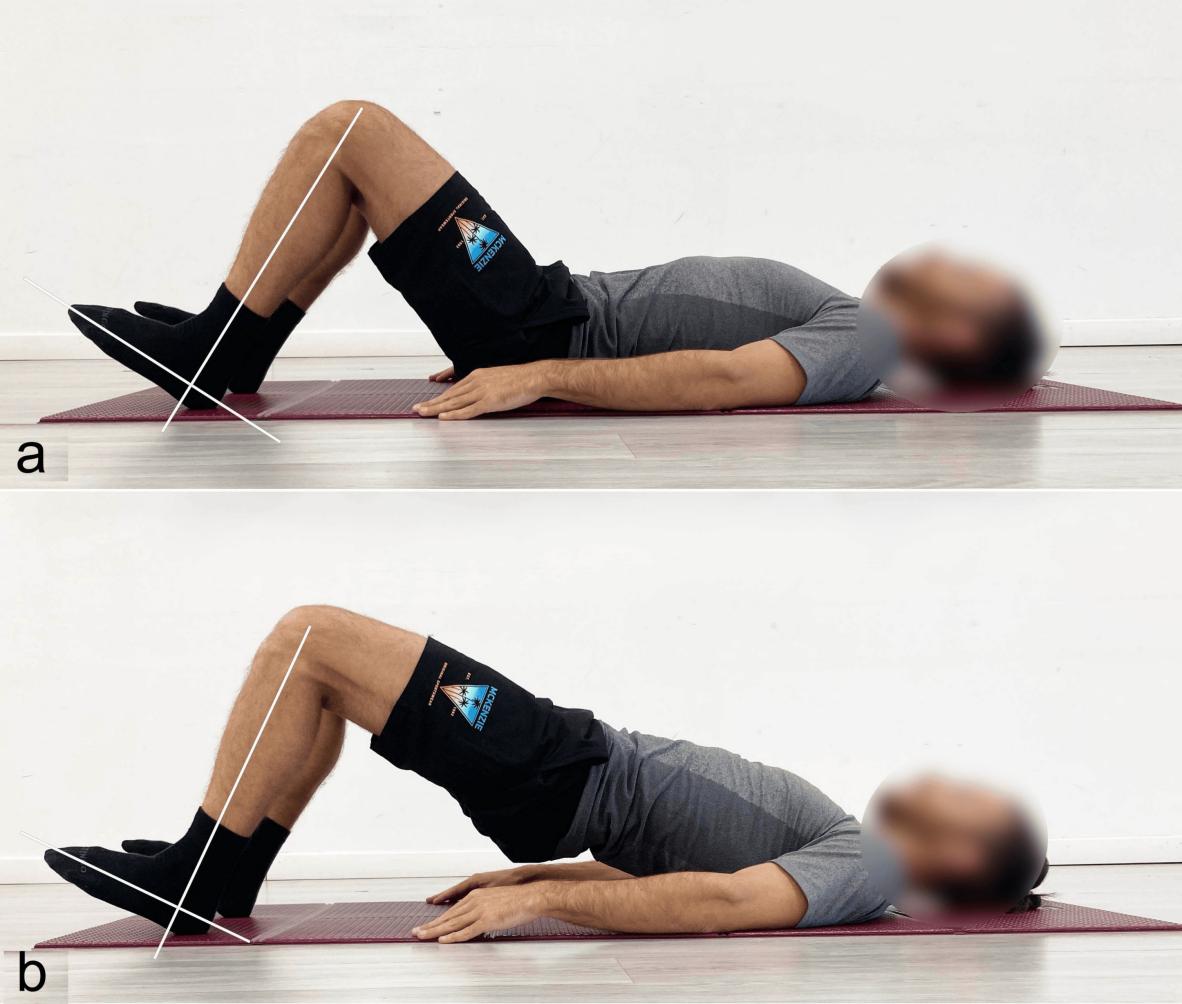
Example of supine bridge exercise performed with ankle dorsiflexion a) Starting position; b) end position. Reproduced from Colonna et al. [4], under a Creative Commons Attribution License (CC BY).

After this lengthy but necessary preamble, it follows that in hip dysfunctions/pathologies, the use of the SBE is highly recommended, especially with significant knee flexion (Figure 6) and ankle dorsiflexion (Figure 7) to reduce hamstring involvement and promote activation of the GM [4].

A separate consideration is needed regarding the positioning of the spine during the exercise in these dysfunctional situations. To provide precise guidelines on this aspect, it is essential to assess the condition of the lumbar curve. Hip dysfunction, caused by abduction/external rotation of the femur, is most commonly associated with spinal flexion dysfunctions [125], which often correlate with a straightening of the lumbar lordosis [125].

**Figure 8:**
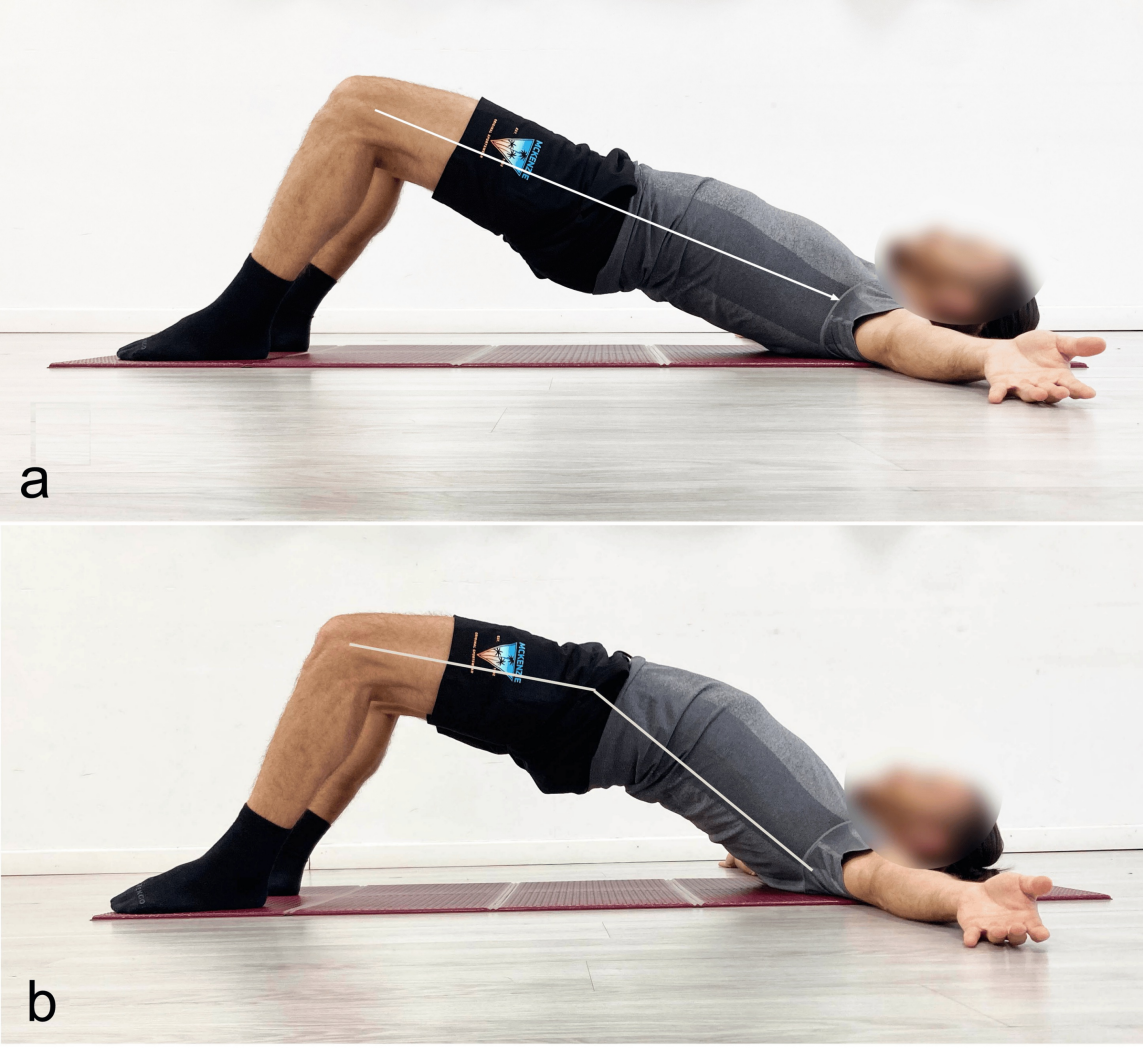
Supine bridge exercise performed with different spinal angles a) Neutral; b) hyperextension. Reproduced from Colonna et al. [4], under a Creative Commons Attribution License (CC BY).

In these cases, it is advisable to activate the sagittal myofascial systems of the lumbar spine, requiring an extension that may reach even maximal angles (Figure 8a). The hip joint extension is one of the most painful movements due to the tension created on the capsule and labrum [32]; therefore, the level of pelvic lift with the corresponding hip extension should not reach excessive pain levels.

**Figure 9:**
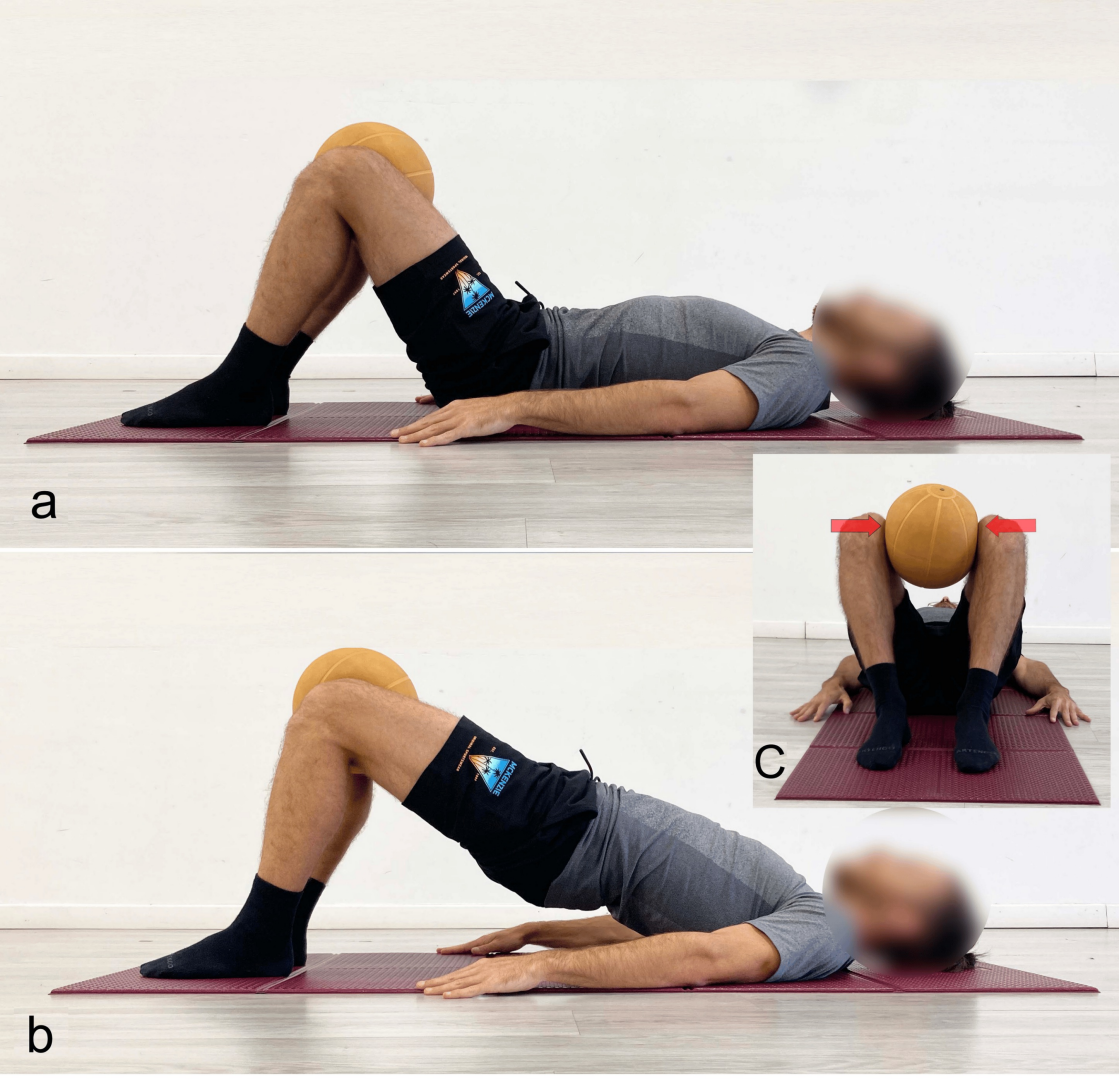
Bridging with the addition of adductor muscle activation using a ball a) Starting position; b) end position, lateral view; c) end position, foot-level view. Reproduced from Colonna et al. [4], under a Creative Commons Attribution License (CC BY).

To inhibit the abductor/external rotator component of the GM, it is recommended to use a ball between the knees (Figure 9) during the bridge exercise to activate the adductors/internal rotators [4].

**Figure 10:**
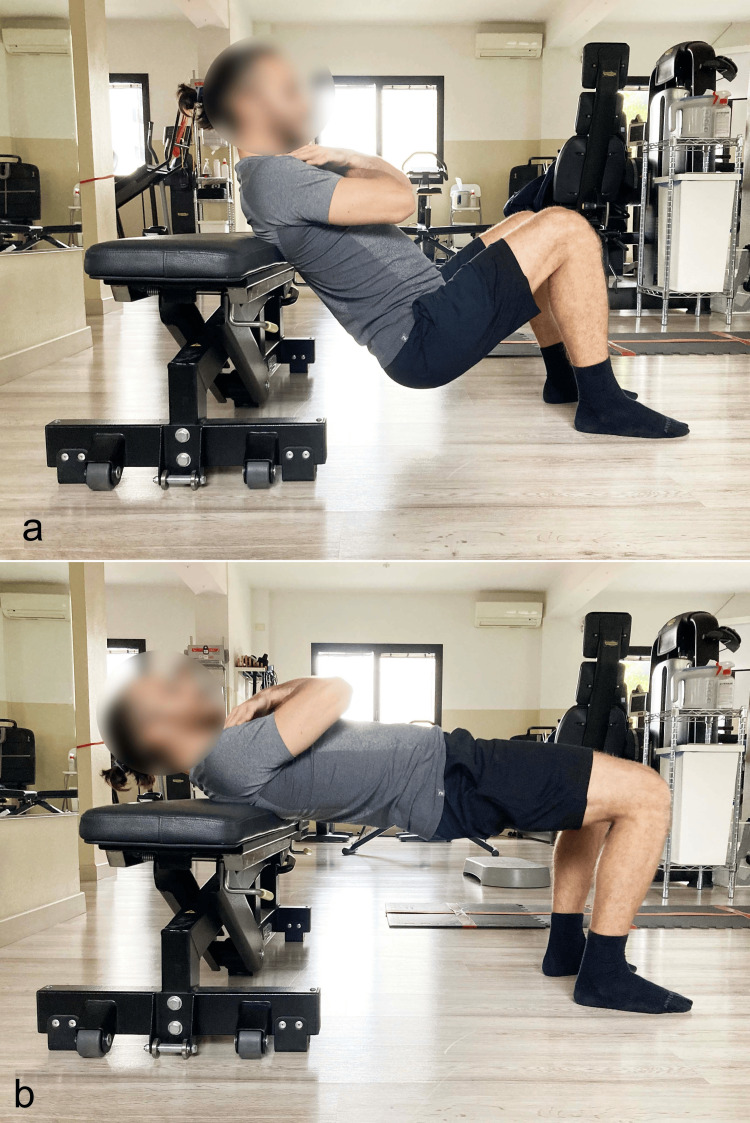
Barbell hip thrust with dorsal support on a bench a) Starting position; b) final position. Reproduced from Colonna et al. [4], under a Creative Commons Attribution License (CC BY).

If there is a hip dysfunction associated with valgus/internal rotation of the lower limb and a lumbar condition with hyperlordosis, referred to as extensor syndrome [40,125], it is recommended, as previously stated during bridging, not to exceed the neutral position of the lumbar spine (Figure 8b). To help maintain a neutral lumbar spine position, it is advised to perform the bridge with the shoulders supported on an elevated surface, approximately the length of the tibia (barbell exercise) (Figure 10).

**Figure 11:**
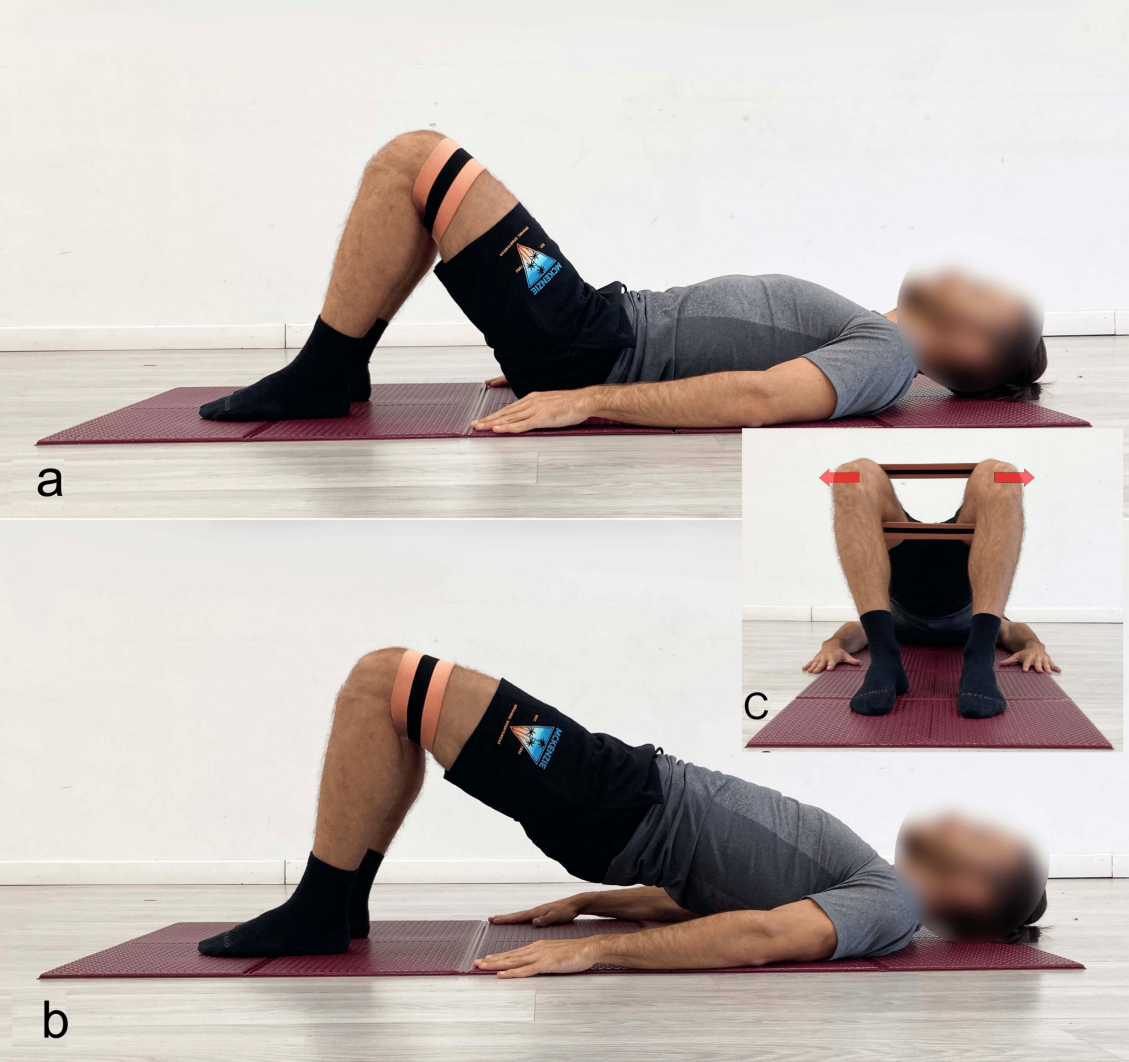
Bridging exercise with added activation of the abductor muscles using an elastic band a) Starting position; b) final position, lateral view; C) final position, foot-level view. Reproduced from Colonna et al. [4], under a Creative Commons Attribution License (CC BY).

**Figure 12:**
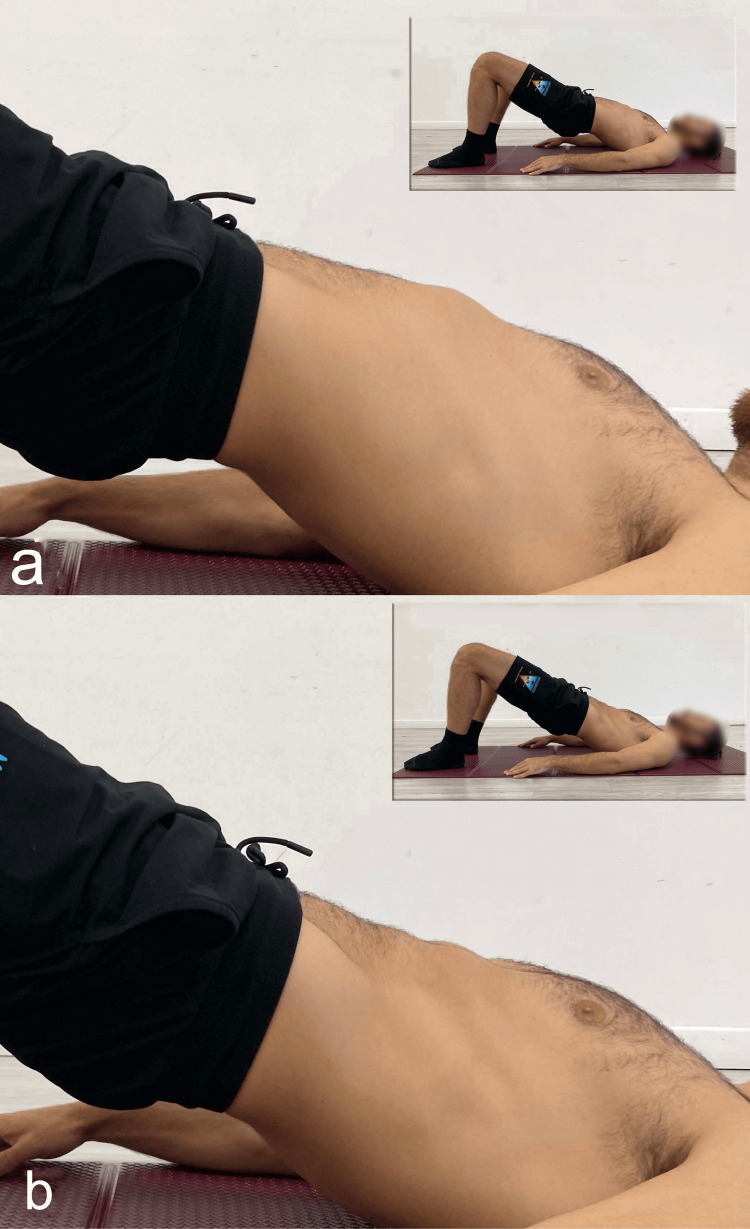
Execution of supine bridge exercise a) Traditional; b) with the abdominal drawing-in maneuver. Reproduced from Colonna et al. [4], under a Creative Commons Attribution License (CC BY).

Additionally, using an elastic band around the knees during the execution of the classic SBE (Figure 11) to activate the abductor/external rotator muscles of the hip and simultaneously contract the abdominal muscles (abdominal drawing-in maneuver) (Figure 12) is recommended.

Regarding the execution modality of the bridge outlined above, we recommend performing it in an isometric condition, at least during the initial sessions. After becoming familiar with the basic execution and focusing the subject/patient's attention on the activation of the GM and deactivation of the hamstrings, slow pelvic lifts can be introduced, maintaining the position for approximately 30 seconds, followed by a slow and controlled return to the starting position. A set consists of three to five repetitions, with a recovery period of one to two minutes, and at least three sets are recommended.

**Figure 13:**
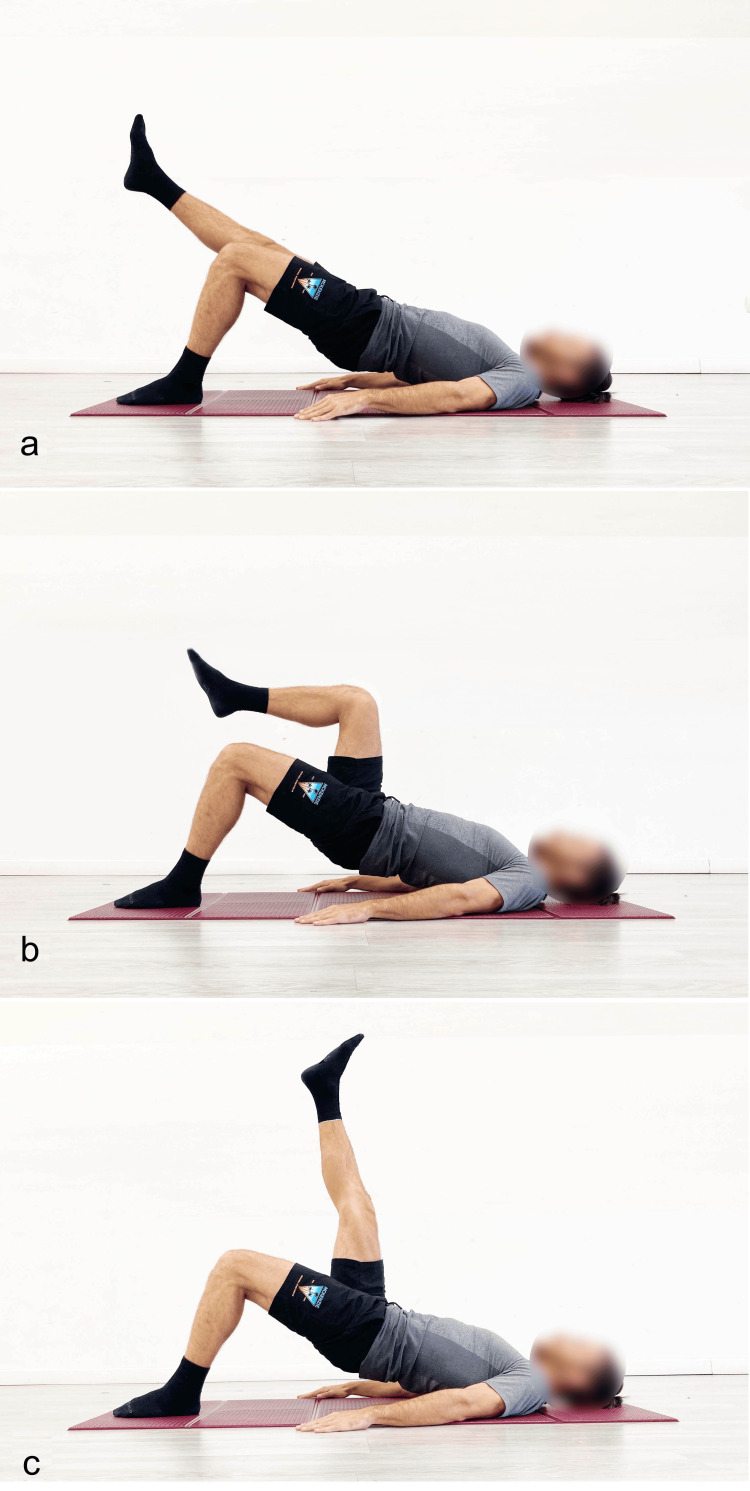
Example of different execution modalities of the single-leg supine bridge exercise a) The limb suspended in line with the trunk; b) hip and knee flexion; c) hip flexion with the knee extended. Reproduced from Colonna et al. [4], under a Creative Commons Attribution License (CC BY).

**Figure 14:**
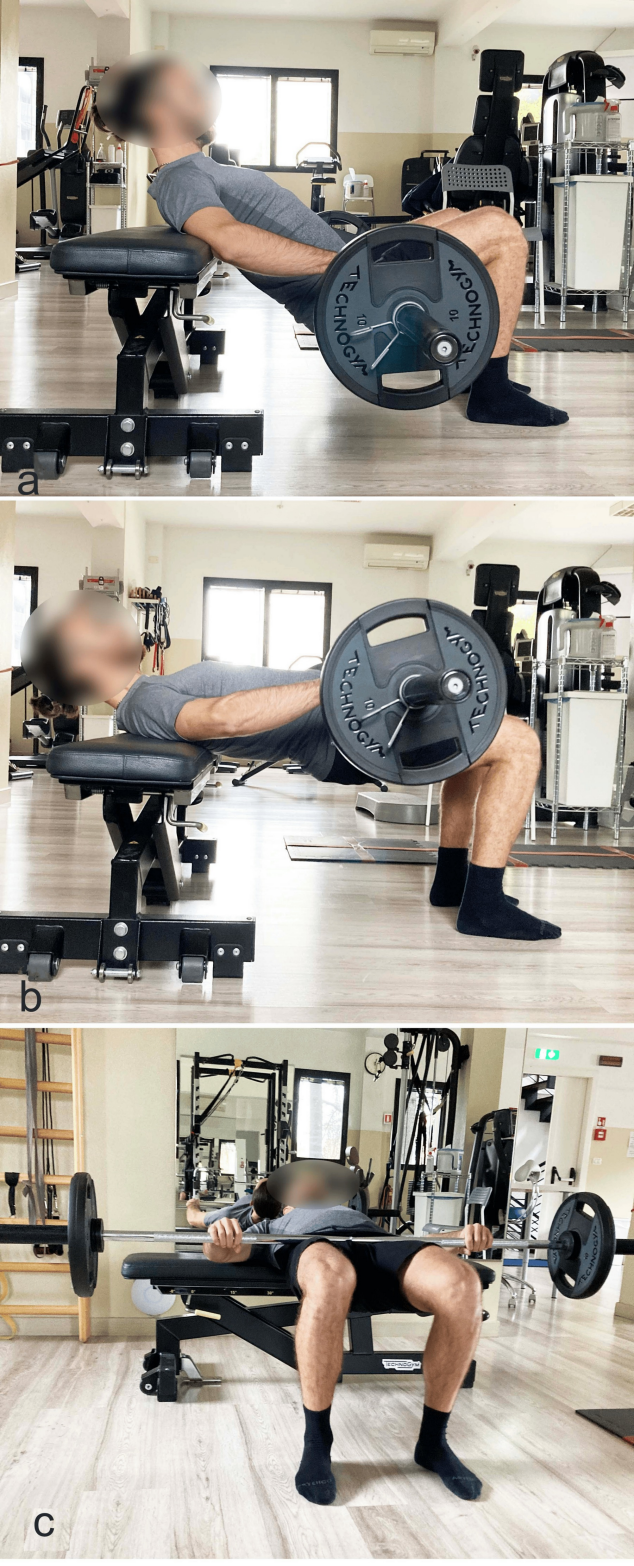
Barbell hip thrust with external load a) Starting position with the barbell; b) end position; c) foot-level view of the end position. Reproduced from Colonna et al. [4], under a Creative Commons Attribution License (CC BY).

There are various ways to increase the load: 1) increase the duration of the pelvic lift position; 2) perform more sets; 3) alternate between bipodal and monopodal execution (pathological limb), without returning to the starting position (20 seconds bipodal, 20 seconds monopodal; 20 seconds bipodal, 20 seconds monopodal) (Figure 13); insert overloads into the barbell hip thrust exercise (Figure 14).

For further details on the technical execution of SBE and its variations, we refer to the first article in this series [4], which discusses these aspects in depth. However, it is important to highlight how these techniques can be applied to specific hip pathologies:

In FAI, the SBE promotes LGM activation and reduces anterior femoral head translation, potentially improving joint mechanics and alleviating impingement-related symptoms. In cases of microinstability, the posterior stabilization provided by LGM activation may help maintain the physiological position of the femoral head, limiting excessive anterior translation and capsular stress. In early osteoarthritis, reducing abnormal shear and compressive forces through improved hip extension mechanics may decrease cartilage load and promote joint preservation. By correcting dysfunctional motor patterns and restoring posterior hip control, SBE may represent a low-load, joint-friendly strategy for neuromuscular retraining and early-stage joint protection.

## Conclusions

In conclusion, the supine bridge exercise (SBE), appropriately individualized based on biomechanical and neuromuscular principles, represents a valuable intervention for addressing anterior femoral glide, enhancing hip joint stability, promoting joint congruency, and restoring muscular balance. Its proper implementation may contribute to slowing the degenerative processes typically observed in non-traumatic hip disorders.

Moreover, given the high prevalence of CAM-type femoroacetabular impingement (FAI) and related hip dysfunctions in specific athletic populations such as soccer players and dancers, the integration of targeted SBE sessions into training and preventive rehabilitation programs is strongly recommended. By selectively activating the lower portion of the gluteus maximus and optimizing femoral head positioning, SBE can play a critical role in preserving hip health, improving functional performance, and potentially reducing the long-term risk of hip osteoarthritis in these at-risk groups.
